# Evaluation of the immune feature of ACPA-negative rheumatoid arthritis and the clinical value of matrix metalloproteinase-3

**DOI:** 10.3389/fimmu.2022.939265

**Published:** 2022-07-27

**Authors:** Zhaojun Liang, Nan Wang, Lili Shang, Yanlin Wang, Min Feng, Guangying Liu, Chong Gao, Jing Luo

**Affiliations:** ^1^ Division of Rheumatology, Department of Medicine, The Second Hospital of Shanxi Medical University, Taiyuan, China; ^2^ Shanxi Key Laboratory for Immunomicroecology, Taiyuan, China; ^3^ Brigham and Women’s Hospital, Harvard Medical School, Boston, MA, United States

**Keywords:** anti-citrullinated protein antibodies, rheumatoid arthritis, lymphocyte subsets, matrix metalloproteinase-3, biomarker

## Abstract

Anti-citrullinated protein antibodies (ACPAs) are highly specific for the diagnosis of rheumatoid arthritis (RA). However, about one-third of RA patients are negative for ACPAs, which presents a challenge to the early diagnosis of RA. The purpose of this study was to analyze differences in lymphocyte subsets and CD4^+^ T cell subsets between ACPA^+^ and ACPA^-^ RA patients, and to evaluate the value of matrix metalloproteinase-3 (MMP-3) as a diagnostic and monitoring marker in ACA^-^ RA patients. A total of 145 ACPA^+^ RA patients, 145 ACPA^-^ RA patients, and 38 healthy controls (HCs) were included in this study. Peripheral lymphocyte subsets were detected using flow cytometry, and serum MMP-3 was detected using chemiluminescence. Information about joint symptoms, other organ involvement, and related inflammatory markers was also collected. The results showed that, compared to ACPA^-^ RA patients, ACPA^+^ cases had greater imbalances between peripheral CD4^+^ T cell subsets, mainly manifested as an increase in T-helper 1 (Th1) cells (*p* < 0.001) and decrease in regulatory T (Treg) cells (*p* = 0.029). This makes these patients more prone to inflammatory reactions and joint erosion. MMP-3 levels in ACPA^+^ and ACPA^-^ RA patients were significantly higher than in HCs (*p* < 0.001), and MMP-3 could effectively distinguish between ACPA^-^ RA patients and HCs (area under the curve [AUC] = 0.930, sensitivity 84.14%, specificity 92.11%). MMP-3 was also a serum marker for distinguishing between RA patients with low and high disease activities. Further analysis showed that MMP-3 was positively correlated with the levels of inflammatory markers and disease activity, and negatively correlated with the levels of lymphocyte subsets. In addition, with improvements in the disease, MMP-3 levels decreased, and further increased as the patients started to deteriorate. In summary, our research showed that there was a mild imbalance between peripheral CD4^+^ T cell subsets in ACPA^-^ RA patients. MMP-3 may be used as a potential marker for early diagnosis of ACPA^-^ RA. MMP-3 was an important index for RA disease evaluation, disease activity stratification, and prognosis.

## Introduction

Rheumatoid arthritis (RA) is an autoimmune disease characterized by synovitis. Persistent synovitis can cause progressive joint injury and functional decline. Early diagnosis is important for preventing disability and maintaining body function. Rheumatoid factor (RF) is a classic autoantibody detectable in 70–80% of RA patients, but can also be present in other autoimmune diseases. About 70–80% of RA patients can produce anti-citrullinated protein antibodies (ACPAs), which are highly specific for RA and are related to the clinical manifestations, treatment, and prognosis of RA patients (1). However, about 1/3 of RA patients are ACPA negative, which makes early diagnosis of RA challenging (2). Studies of differences in lymphocyte and CD4^+^ T cell subsets between ACPA positive (ACPA^+^) and negative (ACPA^-^) RA patients, and identification of new serological markers are of great importance in the early diagnosis of ACPA^-^ RA and in understanding its underlying pathological mechanism. Matrix metalloproteinase-3 (MMP-3) is a proteolytic enzyme that can destroy collagen and proteoglycan, cause abnormal degradation of chondrocyte-dependent extracellular matrix proteins, and lead to cartilage degeneration. In the last 30 years, the role of MMP-3 in RA has been widely studied, but its significance in ACPA- RA has not been reported.

This study included 145 ACPA^+^ RA patients, 145 ACPA^-^ RA patients, and 38 healthy controls (HCs). The distribution of peripheral lymphocyte and CD4^+^ T cell subsets was detected using flow cytometry, and serum MMP-3 content was detected using chemiluminescence. We found a mild imbalance between peripheral CD4^+^ T cell subsets in ACPA^-^ RA patients. MMP-3 may be helpful in the early diagnosis of ACPA^-^ RA and may be used as an index for evaluation, disease activity stratification, and prognosis of RA patients.

## Materials and methods

### Patients and controls

**Figure 1:**
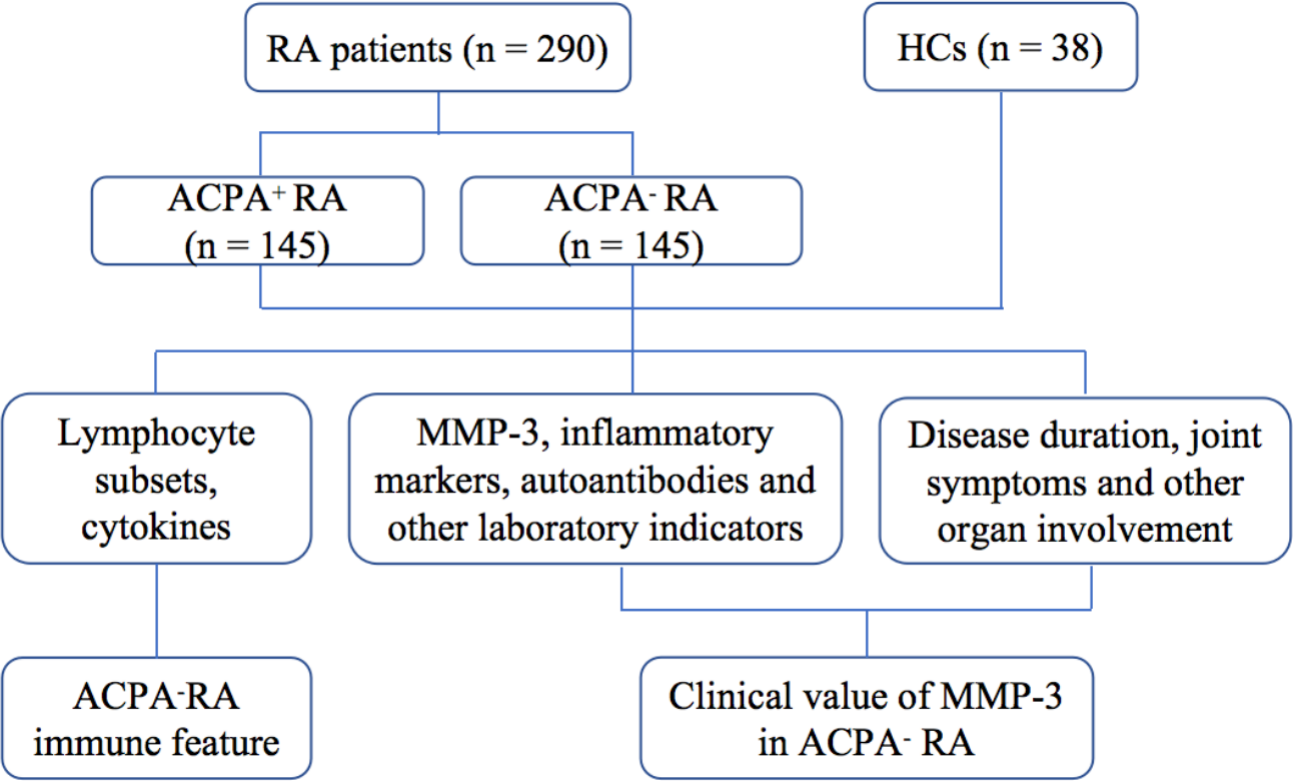
Study design and procedures.

We enrolled 145 ACPA^-^ RA patients between 2016 and 2020, and randomly selected 145 ACPA^+^ RA patients from the same period (**Figure 1**). All patients were newly diagnosed, untreated, and hospitalized in the Department of Clinical Immunology and Rheumatology of the Second Hospital of Shanxi Medical University. The diagnosis was based on the 1987 RA classification diagnostic criteria by the American College of Rheumatology: 1) morning stiffness in and around joints lasting at least 1 hour; 2) at least three soft tissue swellings (arthritis) in the joint area at the same time; 3) swelling (arthritis) of at least one joint area in the proximal interphalangeal, metacarpophalangeal, or wrist joints; 4) symmetric swelling (arthritis); 5) rheumatoid nodules; 6) presence of RF; and 7) X-ray changes (radiographic erosions and/or periarticular osteopenia in hand and/or wrist joints). RA was diagnosed if four of the above seven items were met (criteria 1–4 must be present for at least 6 weeks) (3). Exclusion criteria were severe infections, cancer, and/or other autoimmune diseases. Body mass index (BMI), smoking history, joint symptoms and involvement of other organs were recorded for all RA patients. At the same time, 38 healthy age- and sex-matched subjects, with no family history of autoimmune diseases, were enrolled as the HC group from the physical examination center of the second hospital of Shanxi Medical University. The study was approved by the Ethics Committee of the Second Affiliated Hospital of Shanxi Medical University (number: 2016-KY-014). Written informed consent was obtained from all participants before the study.

### Peripheral venous blood parameters

Venous blood samples were collected from all participants on an empty stomach in the early morning. Routine laboratory tests, including white blood cell (WBC), neutrophil, lymphocyte, erythrocyte sedimentation rate (ESR), C-reactive protein (CRP), vascular endothelial growth factor (VEGF) and RF levels. WBC, neutrophil, and lymphocyte were performed using standard methods in the XN-9000 automatic blood analyzer (Sysmex Corp., Hyogo, Japan). CRP levels were determined using the Beckmann method and the Coulter image 800 automatic protein analyzer (Beckman Coulter Inc., Brea, CA, USA), according to the manufacturer’s instructions. Enzyme-linked immunosorbent assays were used to detect RF, VEGF, immunoglobulin G (IgG), immunoglobulin A (IgA), and immunoglobulin M (IgM). Lymphocyte subsets, including CD4^+^ T cells, CD8^+^ T cells, CD19^+^ B cells, CD16/CD56^+^ natural killer (NK) cells, CD4^+^IFN-γ^+^ T-helper 1 (Th1) cells, CD4^+^IL-4^+^ T-helper 2 (Th2) cells, CD4^+^IL-17^+^ T-helper 17 (Th17) cells, and CD4^+^CD25^+^Foxp3^+^ regulatory T (Treg) cells, were detected using BD-FACS-CANTO II flow cytometry (Becton, Dickinson and Co., Franklin Lakes, NJ, USA) (**Supplementary Figure S1**).

### Analysis of the level of MMP-3

Serum MMP-3 levels were detected in 300 μL serum samples using the automatic chemiluminescence analyzer KAESER 6600 (Kangrun Biotech Co., Ltd., Guangzhou, China) according to the manufacturer’s instructions. Routine operation procedures were referred to in the instrument system operation manual. We used the normal reference ranges for females (17.5–60 ng/mL) and males (26–121 ng/mL) provided in the manual for the MMP-3 detection kit. Those above the normal range were defined as MMP-3 positive and will be used for subsequent result analysis.

### Statistical analysis

All data were analyzed using SPSS 25.0 (IBM Corp., Armonk, NY, USA) or GraphPad Prism 9 (GraphPad Software Inc., San Diego, CA, USA). Normally distributed variables were expressed as means ± standard deviation (M ± SD), nonparametric variables were expressed as medians and interquartile range (IQR), and classified variables were expressed as numbers and percentages. Mann-Whitney U test or independent samples T-test were used to compare two groups, while the Kruskal-Wallis test was used to compare differences among multiple groups. The Chi-square test was used to compare the ratios between two groups. Pearson’s correlation test was used to evaluate the correlation between MMP-3 and other indicators. The areas under the receiver operating characteristic (ROC) curves were used to evaluate the values of serum MMP-3 in different disease states. P < 0.05 was considered statistically significant.

## Results

### Clinical data and characteristics of ACPA^+^ and ACPA^-^ RA patients

A total of 145 ACPA^+^ RA patients (94 females; mean age: 56.93 ± 11.55 years), 145 ACPA^-^ RA patients (109 females; mean age: 56.82 ± 12.87 years), and 38 HCs (26 females; mean age: 51.83 ± 14.13 years) were included in this study (**Table 1**). Compared to ACPA^+^ RA patients, ACPA^-^ RA patients had higher BMIs (*p* = 0.005), but a lower proportion of smoking (*p* = 0.001). The proportion of morning stiffness (*p* = 0.015), joint deformity (*p* = 0.015), and interstitial lung disease (ILD) (*p* = 0.001) in ACPA^+^ RA patients was significantly higher than in ACPA^-^ RA patients, and disease activity score using 28 joint counts (DAS28)-ESR (3) also showed an increasing trend (*p* = 0.120). WBC (*p* = 0.013), ESR (*p* = 0.001), RF positivity (*p* < 0.001) and IgA (*p* = 0.012) levels in ACPA^+^ RA patients were significantly higher than in ACPA^-^ RA patients.

**Table 1:** Demographic characteristics and laboratory values between ACPA^+^ RA, ACPA^-^ RA patients and healthy controls.

Characteristic	ACPA^+^ RA (n = 145)	ACPA^-^ RA (n = 145)	HC (n = 38)	*p* value
Age (years)	56.93 ± 11.55	56.82 ± 12.87	51.83 ± 14.13	0.108
Gender (% female)	94 (64.8)	109 (75.2)	26 (86.7)	0.079
BMI (kg/m^2^)	23.12 ± 3.04	24.20 ± 3.07	24.15 ± 2.21	0.010
Smoker (%)	41 (28.3)	18 (12.4)	6 (15.7)	0.003
Disease duration (years)	6.00 (2.00, 14.25)	4.00 (1.46, 11.00)	–	0.187
Tender joint count	3.00 (1.00, 10.00)	2.00 (0.00, 10.00)	–	0.787
Swollen joint count	6.00 (2.00, 14.00)	8.00 (2.00, 22.00)	–	0.189
DAS28-ESR (3)	5.28 (4.28, 6.32)	5.15 (3.58, 6.20)	–	0.120
Morning stiffness (%)	130 (89.7)	115 (79.3)	–	0.015
Joint deformities (%)	53 (36.6)	27 (18.6)	–	0.001
ILD (%)	30 (20.7)	11 (7.6)	–	0.001
Swollen lymph nodes (%)	38 (26.2)	25 (17.2)	–	0.064
Blood system involvement (%)	28 (19.3)	23 (15.9)	–	0.441
Presence of CVD (%)	53 (36.6)	49 (33.8)	–	0.623
WBC (*10^9^/L)	6.88 (5.72, 8.79)	6.38 (4.86, 8.13)	–	0.013
Neutrophil (*10^9^/L)	4.47 (3.26, 5.87)	3.85 (2.85, 5.69)	–	0.015
Neutrophil (%)	66.10 (59.10, 73.40)	63.40 (55.70, 72.60)	–	0.084
Lymphocyte (*10^9^/L)	1.65 (1.24, 2.01)	1.55 (1.15, 2.17)	–	0.591
Lymphocyte (%)	23.90 (18.00, 30.90)	25.90 (18.70, 33.10)	–	0.103
ESR (mm/h)	57.00 (30.00, 95.00)	39.00 (15.00, 75.00)	–	0.001
CRP (mg/L)	20.35 (7.62, 57.93)	18.60 (4.13, 58.35)	–	0.432
VEGF (mg/ml)	585.05 (314.05, 1006.38)	629.70 (60.80, 931.50)	–	0.804
RF positive (%)	131 (90.3)	16 (11.0)	–	< 0.001
Anti-CCP positive (%)	145 (100)	0	–	–
APF positive (%)	110 (75.9)	0	–	–
AKA positive (%)	111 (76.6)	0	–	–
IgG (g/L)	11.95 (9.54, 14.75)	11.60 (9.05, 15.30)	–	0.480
IgA (g/L)	2.78 (2.07, 3.94)	2.37 (1.78, 3.25)	–	0.012
IgM (g/L)	1.04 (0.73, 1.05)	1.15 (0.82, 1.56)	–	0.369

ACPA, anti-citrullinated protein antibody; RA, rheumatoid arthritis; HC, healthy control; BMI, body mass index; DAS28, disease activity score using 28 joint counts; ILD, interstitial lung disease; CVD, cardiovascular disease; WBC: white blood cells; ESR, erythrocyte sedimentation rate; CRP, C-reactive protein; VEGF, vascular endothelial growth factor; RF, rheumatoid factors; Anti-CCP, anti-cyclic citrullinated peptide antibody; APF, anti-perinuclear factor antibody; AKA, anti-keratin antibody.

### Immune features of ACPA^+^ and ACPA^-^ RA patients

**Figure 2:**
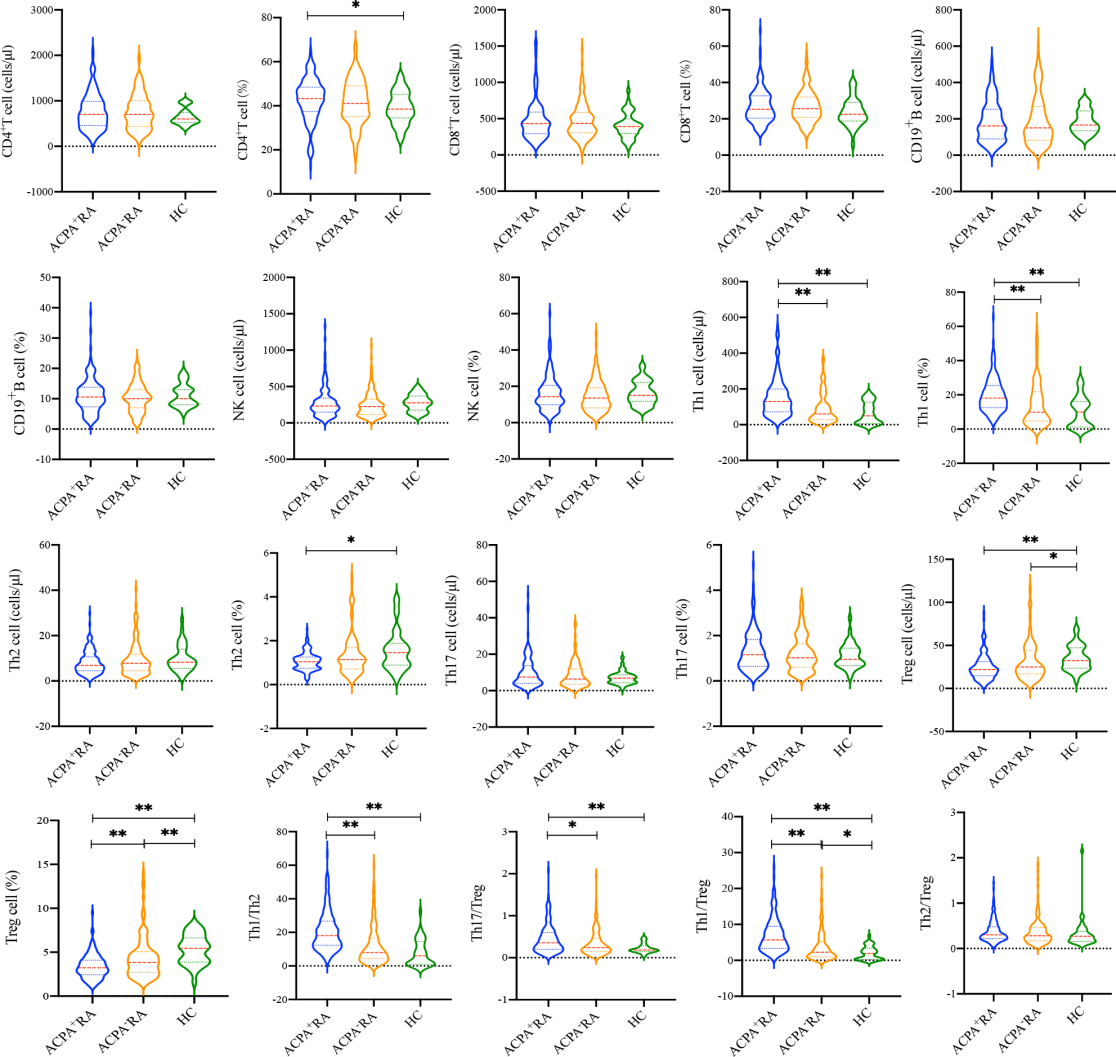
Comparison of the lymphocyte subsets and CD4^+^ T cell subsets between ACPA^+^ RA, ACPA^-^ RA patients and HCs. *p < 0.05, **p < 0.01.

To compare the differences in immune features between ACPA^+^ and ACPA^-^ RA patients, we detected the absolute numbers and percentages of lymphocyte subsets using flow cytometry (**Figure 2**). There were no differences in the absolute number of CD4^+^ T cells among the three groups (*p* = 0.446). The percentage of CD4^+^ T cells in ACPA^+^ RA patients was higher than in HCs (*p* = 0.031), but was similar to ACPA^-^ RA patients (*p* = 0.622). Similarly, there were no significant differences in the absolute number and percentage of CD8^+^ T cells (*p* = 0.366 and 0.113, respectively), CD19^+^ B cells (*p* = 0.608 and 0.682, respectively), and NK cells (*p* = 0.128 and 0.233, respectively) among the three groups. We further analyzed the absolute numbers and percentages of CD4^+^ T cell subsets, including Th1, Th2, Th17, and Treg cells. The absolute number and percentage of Th1 cells in ACPA^+^ RA patients were significantly higher compared to ACPA^-^ RA patients and HCs (*p* < 0.001), while the absolute number and percentage of Treg cells in ACPA^+^ and ACPA^-^ RA patients were lower compared to HCs (*p* < 0.05). Notably, the percentage of Treg cells in ACPA^+^ RA patients was even lower than in ACPA^-^ RA (*p* = 0.003). The Th1/Th2, Th17/Treg, and Th1/Treg ratios were also significantly increased in ACPA^+^ RA patients compared to ACPA^-^ RA patients and HCs (*p* < 0.05).

### MMP-3 levels in RA patients and healthy controls

**Figure 3:**
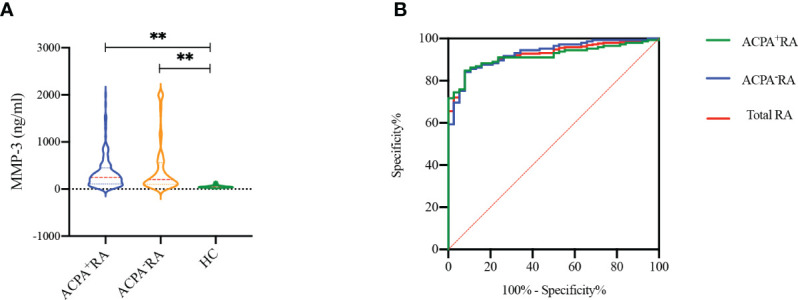
MMP-3 levels between RA patients and healthy controls **(A)**, receiver operating characteristic curves (ROC) of MMP-3 levels to distinguish RA patients from HCs **(B)**. **p >0.01.

Serum MMP-3 levels were detected in RA patients and HCs by chemiluminescence analysis (**Figure 3A**). The results showed that MMP-3 levels in RA patients, including ACPA^+^ and ACPA^-^ patients, were significantly higher than in HCs (*p* < 0.001), but there was no significant difference between the two RA groups (*p* = 0.697). In addition, we evaluated the ability of MMP-3 to distinguish ACPA^+^ RA patients, ACPA^-^ RA patients, and all RA patients from HCs using the ROC curve (**Table 2**; **Figure 3B**). We found that MMP-3 had a similar ability to distinguish between the three RA groups and HCs: the AUCs were 0.917 (95% confidence interval [CI] 0.877–0.957; *p* < 0.01), 0.930 (95% CI 0.892–0.968; *p* < 0.01), and 0.923 (95% CI 0.891–0.956; *p* < 0.01), respectively.

**Table 2:** Diagnostic accuracy of MMP-3 in RA patients.

	AUC(95% CI)	Cut-off	Prediction RA (%)		+LR	−LR	PPV	NPV
			Sensitivity	Specificity				
ACPA^+^ RA	0.917 (0.877, 0.957)	78.95	84.83	92.11	10.75	0.16	97.62	61.40
ACPA^-^ RA	0.930 (0.892, 0.968)	78.79	84.14	92.11	10.66	0.17	97.60	60.34
Total RA	0.923 (0.891, 0.956)	78.79	84.48	92.11	10.71	0.17	98.79	43.75

MMP-3, matrix metalloproteinase-3; CI, confidence interval; AUC, area under the curve; +LR, positive likelihood ratio; −LR, negative likelihood ratio; PPV, positive predictive value; NPV, negative predictive value.

### Comparison of MMP-3 and other examined markers in RA patients

**Figure 4:**
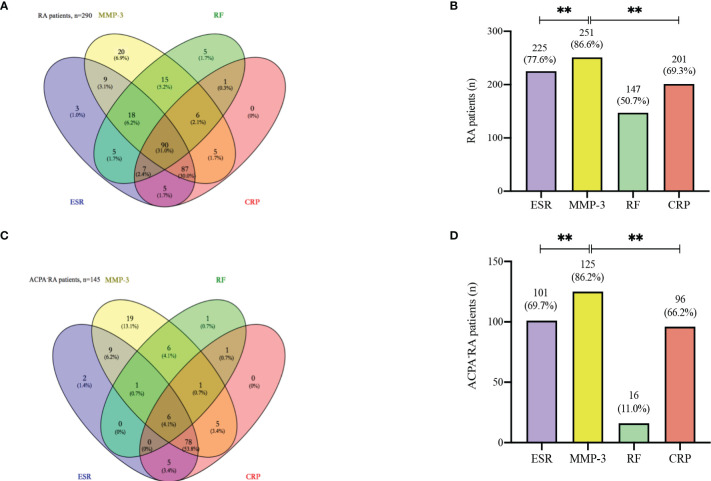
Venn diagram. **(A)** Venn diagram with number and percentage of positive results from the whole 290 RA patients. **(B)** The positive rate of each index in 290 RA patients. **(C)** Venn diagram with number and percentage of positive results from the 145 ACPA^-^ RA patients. **(D)** The positive rate of each index in 145 ACPA^-^ RA patients. ** *p* < 0.01.

We constructed the Wayne diagram of the four examined markers for RA patients: MMP-3, RF, ESR, and CRP. All four markers were found to be positive in 90 of the 290 RA patients (32.5%) (**Figure 4A**). RF was less effective than MMP-3 as a marker for RA. We also compared the positivity rates of the four markers in RA patients, and found that the positivity rate of MMP-3 was significantly higher than that of other makers (**Figure 4B**). This finding was also observed in ACPA^-^ RA patients (**Figures 4C**, **D**).

### Stratification of disease activity using MMP-3

**Figure 5:**
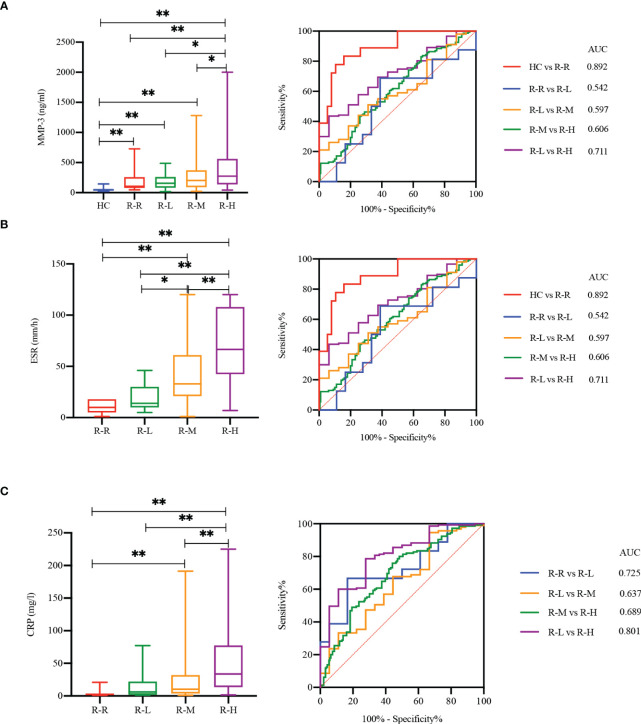
MMP-3 **(A)**, ESR **(B)** and CRP **(C)** correlated with RA disease activity. Boxplots and ROC analysis of the level differences of four markers between HC, RA with remission stage [R-R, DAS28-ESR (3) < 2.6], low disease activity [R-L, DAS28-ESR (3) ≤ 3.2], moderate disease activity [R-M, 3.2 < DAS28-ESR (3) ≤ 5.1] and high disease activity [R-H, DAS28-ESR (3) > 5.1]. *p < 0.05, **p < 0.01.

We stratified the RA patients using disease activity score DAS28-ESR (3), and analyzed the practicability of MMP-3 for RA case stratification based on disease activity (**Figure 5**). MMP-3, ESR, and CRP levels were positively correlated with disease activity. MMP-3 levels in remission and in low, medium, and high disease activity groups were significantly higher than those in the HC group (*p* < 0.001). MMP-3, ESR, and CRP in the high disease activity group were significantly higher than those in the low disease activity group (*p* < 0.05, AUC > 0.7), indicating their potential as biomarkers in RA disease activity stratification.

### Comparison of clinical characteristics between MMP-3 positive and negative RA patients

**Figure 6:**
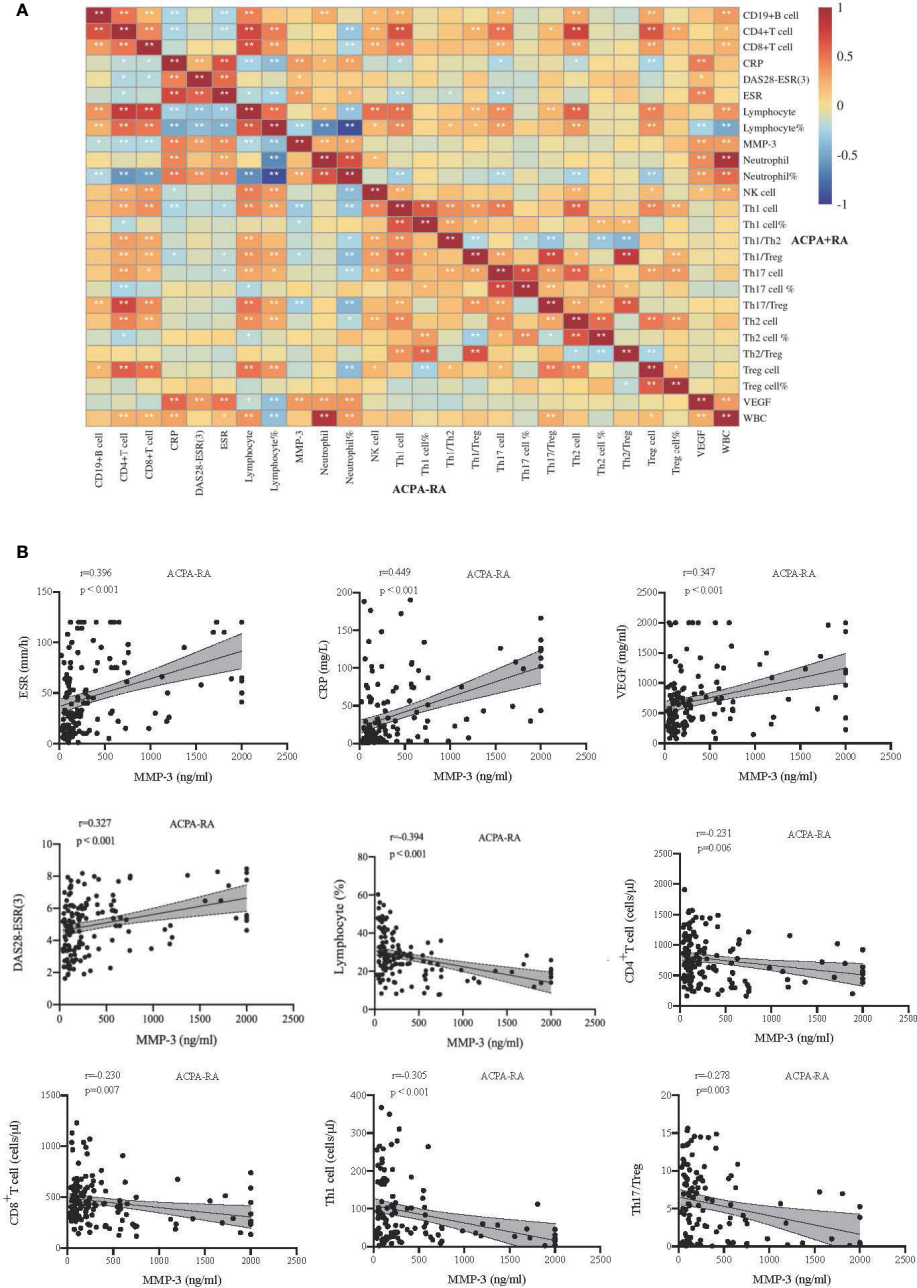
Correlations between indicators in RA subgroup. **(A)** The upper right corner and lower left corner of the heat map show the correlation of inflammatory markers and lymphocyte subsets in patients with ACPA^+^, ACPA^-^, respectively. **(B)** Correlation analysis between MMP-3 and some indexes in ACPA^-^ patients. *p < 0.05, **p < 0.01.

We compared the clinical characteristics of MMP-3 positive and MMP-3 negative RA and total RA (Supplementary Table S1), and found that among ACPA^+^ RA patients, MMP-3 positive patients were more likely to have morning stiffness (*p* < 0.001). MMP-3 positive RA patients had greater disease activity (*p* < 0.05), and inflammatory markers, such as ESR (*p* < 0.01), CRP (*p* < 0.001), and VEGF (*p* < 0.05), were also significantly raised regardless of ACPA positivity, which was consistent with the results of the correlation analysis (**Figure 6**). Meanwhile, neutrophil and lymphocyte percentages in MMP-3 positive patients were also higher than those in MMP-3 negative patients (*p* < 0.05). Overall, lymphocyte subset levels in MMP-3 negative RA patients were higher than those in MMP-3 positive RA patients, especially CD19^+^ B cells, NK cells, Th17 cells, and Treg cells.

### Correlations between MMP-3 levels and inflammatory markers in ACPA^-^ RA patients

We analyzed the correlation between MMP-3 levels and systemic inflammatory markers, CD4^+^ T cell subsets, and DAS28-ESR (3) scores in ACPA^+^, ACPA^-^, and total RA patients. In ACPA^-^ RA, MMP-3 was positively correlated with ESR (r = 0.396, *p* < 0.001), CRP (r = 0.449, *p* < 0.001), VEGF (r = 0.347, *p* < 0.001), and DAS28-ESR (3) (r = 0.327, *p* < 0.001), and negatively correlated with the lymphocyte percentage (r = −0.394, *p* < 0.001), CD4^+^ T cells (r = −0.231, *p* = 0.006), CD8^+^ T cells (r = −0.230, *p* = 0.007), Th1 cells (r = −0.305, *p* < 0.001) and Th17/Treg ratio (r = −0.278, *p* = 0.003) (**Figures 6A**, **B**). In ACPA^+^ RA, MMP-3 was positively correlated with ESR (r = 0.314, *p* < 0.001), CRP (r = 0.344, *p* < 0.001), and VEGF (r = 0.344, *p* < 0.001) and negatively correlated with the lymphocyte percentage (r = −0.260, *p* = 0.002) (**Figure 6A**, **Supplementary Figure S2B**). In total RA, MMP-3 was positively correlated with ESR (r = 0.336, *p* < 0.001), CRP (r = 0.400, *p* < 0.001) and VEGF (r = 0.319, *p* < 0.001), and negatively correlated with the lymphocyte percentage (r = −0.328, *p* < 0.001) (**Supplementary Figures S2A**, **C**).

### MMP-3 may be a marker to judge the prognosis of RA

We monitored the changes in MMP-3, DAS28-ESR (3), ESR, CRP, VEGF and lymphocyte subsets in 32 RA patients. Patients were divided into improvement (n = 16) and progression (n = 16) groups based on the number and degree of joint swellings. MMP-3 decreased in the improvement group, along with significant decreases in DAS28-ESR (3) scores, ESR and CRP (**Supplementary Figure S3A**). Meanwhile, MMP-3, ESR and CRP increased in the progression group (**Supplementary Figure S3B**), along with a significant increase in the DAS28-ESR (3) score, but there were no significant differences in CD4^+^ T cells, CD8^+^ T cells, Th17 cells and Treg cells. A total of 32 patients were treated with 0–10 mg prednisone, 10 mg leflunomide once a day, or 250 mg sirolimus once every other day for 13 (4, 5) months.

## Discussion

ACPAs are a series of antibodies, including anti-perinuclear factor (APF), antikeratin antibody (AKA), anti-cyclic citrullinated peptide antibody (CCP), and anti-mutated citrullinated vimentin (MCV) among others (6). ACPAs can be present in the sera of the individuals years before the clinical diagnosis, and the titers increase as individuals approach clinical RA (7, 8). The specificity of ACPAs increases with the number of ACPA subtypes in a time-dependent manner, and the affinity of ACPAs increases over time until disease onset (4, 9). This indicates that preclinical RA involves the accumulation of multiple autoantibody specificity, while the later stages involve the expansion of the antibody response. The expansion of the ACPA response strongly predicts the increase of many inflammatory cytokines, including tumor necrosis factor- α (TNF-α), IL-6, IL-12p70 and interferon gamma (IFN-γ).

ACPA^-^ RA accounts for about 30% of the RA patients, which presents a diagnostic challenge. The diagnosis may be missed and the treatment delayed, resulting in irreversible joint injury. At present, it is clear that ACPA^-^ RA is less prone to bone destruction than ACPA^+^ RA (10). Harre and colleagues confirmed that the presence of ACPAs can increase bone resorption in RA patients. *In vitro* cell research has demonstrated that ACPAs can increase the generation and activation of osteoclasts, resulting in increased bone resorption (11). In our study, we found that the incidence of morning stiffness and joint deformity was much lower in ACPA^-^ RA compared to ACPA^+^ RA. We also found that the probability of pulmonary interstitial fibrosis in ACPA^-^ RA was lower, which may be related to the lack of ACPAs *in vivo*, although there are few reports on the relationship between them.

Genetic, environmental and autoimmune factors play a key role in RA pathogenesis. The heritability of RA has been reported as 50–60% (12), and human leukocyte antigen-DR (HLA-DR), protein tyrosine phosphatase non-receptor 22 (PTPN22), tumor necrosis factor receptor-associated factor 1/complement component 5 (TRAF1/C5) and peptidyl arginine deiminase 4 (PADI4) alleles have been implicated in the genetic predisposition. HLA-DR is a major histocompatibility complex cell-surface receptor that interacts with T-cell receptors by presenting internalized antigens, and stimulates T and B cells. HLA-DRB1 alleles share a common amino acid sequence, i.e. a shared epitope. Glutamine or arginine at the 70^th^ position of the shared epitope is important for the occurrence of RA, while aspartic acid at this position seems to have a protective effect, which not only reduces the risk of RA but also reduces its severity. In addition to RA susceptibility, the shared epitope is also considered to promote joint destruction and extraarticular involvement (13). PTPN22 is considered one of the strongest genetic risks for the development of autoimmunity, with the exception of the HLA region. Changes in PTPN22 may affect both the quantity and quality of the T cell immune response, and may increase the risk of autoimmune diseases. PTPN22 is one of the highest confidence direct targets of Foxp3. Inhibiting the activation of T cell stimulation by target genes is crucial to the normal function of Treg cells (14). NK cells express a high level of PTPN22, which plays an important immunomodulatory role in NK cell function by affecting T cell-dependent NK cell expansion (15). TRAF1/C5 is related to the occurrence and development of inflammation, and has a certain value in predicting RA severity, such as the degree of joint destruction. TRAF1/C5 mutations may lead to changes in the structure, function and expression level of the gene, and thus affect RA severity (16). PADI4 encodes a catalytic enzyme involved in the post-translational modification of arginine residues to citrulline. Increased PADI4 stability leads to the accumulation of citrullinated proteins, and enhances the production of autoantibodies against citrullinated peptides (17). It has been reported that the PADI4 gene is positively correlated with RA, and is one of the genes of RA susceptibility (18).

Apart from genetic factors, RA occurrence and development is largely due to the disorder of immune function (19). Lymphocytes can be divided into T cells, B cells, and NK cells based on their cell surface markers and functions. B cells produce immunoglobulin, and can differentiate into plasma cells to produce antibodies following antigen stimulation (20). In RA, B cells are highly efficient antigen-presenting cells that selectively ingest and present antigens through specific B cell receptors, cause T lymphocyte activation, and produce an autoimmune response (21). In addition, activated B cells can also secrete a variety of pro-inflammatory cytokines, such as transforming growth factor beta (TGF-β), IL-4, IL-6 and IFN-γ, leading to inflammation and joint destruction. NK cells play an important role in innate immunity (22). In this study, the immune characteristics of ACPA^+^ and ACPA^-^ RA were analyzed based on peripheral lymphocyte subsets. There were no significant differences in B and NK cell levels among ACPA^+^ RA, ACPA^-^ RA, and HCs. Therefore, RA development may not only depend solely on cellular and innate immunity but also on humoral immunity.

CD4^+^ T helper cells are a lymphocyte subset that plays an important role in specific immune responses of the body (23). Research has confirmed that an imbalance in CD4^+^ T helper cell subsets is an important reason for RA occurrence and development (24). Under antigen stimulation, based on the microenvironment of different cytokines, naive CD4^+^ T helper cells can differentiate into at least four Th cell subsets: Th1, Th2, Th17 and Treg cells. Early studies have reported an imbalance in the number and function of Th1 and Th2 cells in RA patients (25). It has also been demonstrated that the number of Th17 cells in the peripheral blood of RA patients increases significantly and is closely related to disease activity, while the number and function of Treg cells decreases, resulting in a Th17/Treg imbalance (5, 26). This study demonstrated that there were no differences in the absolute number and percentage of CD4^+^ T cells between ACPA^+^ and ACPA^-^ RA patients, which was consistent with the results of Sandra et al. (27). Compared to ACPA^+^ RA, the absolute number and percentage of Th1 cells and Th1/Th2 ratio in the peripheral blood of ACPA^-^ RA patients were significantly lower. In addition, the absolute number and percentage of Treg cells in the peripheral blood of ACPA^-^ RA patients were significantly increased, and Th17/Treg ratio was significantly decreased. This suggests that there are differences in the CD4^+^ T cell subsets between ACPA^+^ and ACPA^-^ RA. ACPA^+^ RA patients show more serious immune imbalances, which may accelerate disease progression and cause bone erosion.

To realize the functions of diagnosis, prediction and prognosis, the selection and optimization of biomarkers are of great significance. As mentioned previously, although ACPAs have a high specificity in RA diagnosis, they are negative in about 1/3 of the patients. RF titers can be used as an index for RA severity, but it is not unique to RA. ESR and CRP have high sensitivity in evaluating RA disease activity. These indicators have limitations as biomarkers for RA, especially ACPA^-^ RA (28). MMP-3 is an important member of the metalloproteinase family, mainly secreted by chondrocytes and fibroblast-like synovial cells. MMP-3 is involved in joint destruction in RA patients by degrading type II, III, IV, IX, and X collagen, proteoglycan, fibronectin, laminin and elastin (29). Vascular invasion is one of the signs of cartilage degeneration, and MMP-3 is involved in pannus invasion and cartilage degradation (30). MMP-3 is not directly involved in the progression of synovitis, but the products of cartilage matrix decomposition, such as cartilage oligomeric matrix proteins and damage-related molecular patterns, can recruit inflammatory cells and lead to synovitis (31). Apart from directly acting on extracellular matrix components, MMP-3 also plays an important role in the destruction of connective tissue by activating procollagenase. The importance of MMP-3 in RA pathogenesis is reflected in its level, in the synovial fluid in synovitis (32). Serum MMP-3 levels have diagnostic value as laboratory markers for RA (33). Its systemic levels significantly reflect the activation of histological grade, score, and synovial matrix sub-score (33).

MMP-3 increases early in RA (symptoms < 1 year), and shows an increasing trend with disease progression (34). Early studies have shown that MMP-3 levels are associated with aggressive disease, and have a strong correlation with Larsen score progression in patients without joint erosion (35). In a study by Martina et al., MMP-3 levels in RA patients were significantly higher than in HCs, and were positively correlated with ESR, CRP, DAS28-3 and anti-CCP (36). Hu and colleagues also confirmed that in early RA patients with normal CRP and/or ESR, the positivity rate of serum MMP-3 was higher than that of CRP and ESR, and that it was positively correlated with CRP levels (37). Recently, Zhang et al. applied single-cell RNA sequencing to characterize the cell composition and gene expression signature of CD45+ cells in the synovial membranes of ACPA^-^ RA patients. They found that the MMP-3 gene was significantly up-regulated in dendritic cells, macrophages, and T cell subsets in the synovial tissues of ACPA^-^ RA patients (38). We detected the serum MMP-3 levels in ACPA^+^ and ACPA^-^ RA patients, and used HCs for comparison. It was found that there was no significant difference in MMP-3 levels between the two RA groups, but they were significantly higher than in HCs. ROC curve analysis demonstrated that MMP-3 had no significant differences in the ability to distinguish ACPA^+^, ACPA^-^ and all RA patients from HCs. In addition, the Wayne diagram demonstrated that in ACPA^-^ RA, the MMP-3 positivity rate was significantly higher than that of ESR, CRP and RF. Therefore, MMP-3 may be helpful for RA diagnosis, especially in serum ACPA^-^ RA. Surprisingly, RF was not as useful as MMP-3, as a marker for RA, and the addition of MMP-3 could be more beneficial.

Compared to its diagnostic ability, MMP-3 was more useful for evaluating RA disease activity. Shen and colleagues studied RA patients in different disease states, and found that MMP-3 was closely related to the DAS28, and increased with the RA severity. Low MMP-3 levels in RA patients may indicate that the condition is relatively stable (39). Hattori demonstrated that the accuracy of serum MMP-3 levels in predicting clinical remission was higher than that of CRP. Normal MMP-3 levels, combined with CRP levels and disease activity, were helpful in predicting clinical remission and normal physical function in RA patients (40). We analyzed the correlation between MMP-3 levels and inflammatory markers in the RA subgroups and total RA patients. There was a significant correlation between MMP-3 levels and inflammatory markers, such as ESR, CRP, and VEGF, in ACPA^-^ RA, indicating that MMP-3 was involved in the inflammatory response in RA, and was related to the intensity of inflammation. MMP-3 was also positively correlated with the DAS28-ESR (3) score, indicating that MMP-3 may be used as an index for disease activity. MMP-3 levels may be used for RA disease activity stratification to distinguish between patients with low and high disease activity.

MMP-3 may also be used as a prognostic indicator. According to a study by Zhou et al., serum MMP-3 levels in RA patients were significantly higher than in HCs, and were significantly higher in moderate and severe RA than in stable RA. MMP-3 levels and US7 scores decreased significantly in the second week after treatment with certolizumab pegol (Cimzia; UCB, Brussels, Belgium), which confirmed that serum MMP-3 levels and US7 scores could reflect the disease activity and treatment response in moderate and severe RA (41). Takemoto also suggested that the reduction of MMP-3 levels was key to predicting the clinical efficacy of abatacept (42). In a study of methotrexate treatment in RA, low MMP-3 levels were found to be an indicator of lack of progression of joint erosion (43). We also followed up 32 RA patients and found that, with improvement in the disease, DAS28-ESR (3) scores and ESR, CRP and MMP-3 levels decreased, while MMP-3 levels further increased with subsequent disease progression. Therefore, these laboratory markers may be used as prognostic indicators for RA and to monitor treatment effectiveness.

In summary, MMP-3, a member of the MMP family, has significance in ACPA^-^ RA. It may be an indicator for disease monitoring and prognosis evaluation in RA, and may even constitute a potential therapeutic target, which is a good research prospect.

Some limitations of this study should be mentioned. First, the sample size of ACPA^+^ patients and HCs were relatively small. Second, continuous monitoring data of patients during the disease was limited; this requires further verification with more patients.

## Conclusion

This study showed that ACPA^-^ RA patients have a certain degree of immune imbalance, but not as serious as ACPA^+^ RA patients. MMP-3 had greater significance in ACPA^-^ RA patients. It may be used as a marker to assist in the diagnosis of ACPA^-^ RA. It was also an important index for the disease evaluation, stratification of disease activity, and prediction of clinical progression in ACPA^-^ RA patients.

## Data Availability Statement

The original contributions presented in the study are included in the article/**Supplementary Material**. Further inquiries can be directed to the corresponding author.

## Ethics Statement

The studies involving human participants were reviewed and approved by the ethics committee of the Second Affiliated Hospital of Shanxi Medical University. The patients/participants provided their written informed consent to participate in this study.

## Author Contributions

ZL and NW conducted the experiments, analyzed the data and drafted the manuscript. LS and GL collected clinical data. YW and MF collected and stored the samples. CG reviewed and edited the manuscript. JL conceived the topic and revised the content of the manuscript. All authors contributed to the article and approved the submitted version.

## Funding

This work was supported by the Nature Fund Projects of Shanxi Science and Technology Department (201901D111377), the Scientific Research Project of Health commission of Shanxi Province (2019044), the Research Project Supported by Shanxi Scholarship Council of China (2020-191) and the Science and Technology Innovation Project of Shanxi Province (2020SYS08)

## Conflict of Interest

The authors declare that the research was conducted in the absence of any commercial or financial relationships that could be construed as a potential conflict of interest.

## Publisher’s Note

All claims expressed in this article are solely those of the authors and do not necessarily represent those of their affiliated organizations, or those of the publisher, the editors and the reviewers. Any product that may be evaluated in this article, or claim that may be made by its manufacturer, is not guaranteed or endorsed by the publisher.
