# Supplementary material for: Evaluation of the immune feature of ACPA-negative rheumatoid arthritis and the clinical value of matrix metalloproteinase-3
Source: Front Immunol. 2022 Jul 27;13:939265. doi: 10.3389/fimmu.2022.939265 (PMC9363571; doi:10.3389/fimmu.2022.939265)
Supplement: Supplementary file 1 [file DataSheet_1.docx]

Supplementary Material

**Supplementary Table S1.** Comparison of clinical characteristics between MMP-3 positive and negative patients with RA.

|  | ACPA^+^ RA |  |  | ACPA^-^ RA |  |  | Total RA |  |  |
| --- | --- | --- | --- | --- | --- | --- | --- | --- | --- |
|  | MMP-3^+^  (n = 126) | MMP-3^-^  (n = 19) | *p* value | MMP-3^+^  (n = 125) | MMP-3^-^  (n = 20) | *p* value | MMP-3^+^  (n = 251) | MMP-3^-^  (n = 39) | *p* value |
| Tender joint count | 6.50  (3.00, 14.00) | 3.00  (1.00, 10.00) | 0.109 | 6.00  (2.00, 20.00) | 4.50  (2.50, 14.00) | 0.796 | 6.00  (2.00, 16.00) | 4.00  (1.00, 14.00) | 0.247 |
| Swollen joint count | 3.00  (1.00, 10.00) | 2.00  (0, 10.00) | 0.248 | 2.00  (0, 10.00) | 0  (0, 7.00) | 0.090 | 3.00  (0, 10.00) | 1.00  (0, 8.00) | 0.048 |
| DAS28-ESR(3) | 5.31  (4.42, 6.42) | 3.94  (3.61, 5.87) | 0.023 | 5.24  (3.66, 6.21) | 3.96  (3.13, 5.28) | 0.045 | 5.29  (4.18, 6.32) | 3.94  (3.33, 5.68) | 0.003 |
| Morning stiffness (%) | 115 (91.3) | 15 (78.9) | ＜ 0.001 | 98 (78.4) | 15 (75.0) | 0.960 | 213 (84.9) | 30 (76.9) | 0.211 |
| Joint deformities (%) | 48 (38.1) | 5 (26.3) | 0.320 | 23 (18.4) | 4 (20.0) | 1.000 | 71 (28.3) | 9 (23.1) | 0.498 |
| ILD (%) | 29 (23.0) | 1 (5.3) | 0.140 | 10 (8.0) | 1 (5.0) | 0.987 | 39 (15.5) | 2 (5.1) | 0.083 |
| Presence of CVD (%) | 47 (37.3) | 6 (31.6) | 0.629 | 46 (36.8) | 4 (20.0) | 0.142 | 93 (37.1) | 10 (25.6) | 0.166 |
| WBC (*10^9^/L) | 7.11  (6.01, 8.86) | 5.52  (4.79, 7.26) | 0.007 | 6.42  (5.15, 8.27) | 5.55  (4.59, 7.13) | 0.127 | 6.72  (5.43, 8.53) | 5.52  (4.72, 7.17) | 0.003 |
| Neutrophil (*10^9^/L) | 4.69  (3.73, 6.13) | 2.99  (2.46, 3.89) | ＜ 0.001 | 3.96  (3.05, 5.72) | 2.95  (2.33,4.70) | 0.040 | 4.38  (3.35, 5.79) | 2.99  (2.36, 4.31) | ＜ 0.001 |
| Neutrophil (%) | 68.45  (61.13, 73.95) | 54.30  (48.90, 62.90) | ＜ 0.001 | 63.75  (56.50, 72.53) | 62.50  (40.90, 73.24) | 0.112 | 65.70  (58.93, 73.68) | 56.30  (44.63, 65.95) | ＜ 0.001 |
| Lymphocyte (*10^9^/L) | 1.65  (1.23, 1.99) | 1.68  (1.29, 2.40) | 0.584 | 1.52  (1.12, 2.09) | 1.69  (1.33, 2.64) | 0.148 | 1.61  (1.15, 2.01) | 1.68  (1.30, 2.41) | 0.144 |
| Lymphocyte (%) | 4.69  (3.73, 6.13) | 2.99  (2.46, 3.89) | ＜ 0.001 | 3.96  (3.05, 5.72) | 2.95  (2.33, 4.70) | 0.040 | 4.38  (3.35, 5.79) | 2.99 (2.36, 4.31) | ＜ 0.001 |
| ESR (mm/h) | 61.00  (33.75, 98.75) | 30.00  (18.00, 55.00) | 0.002 | 46.00  (20.50, 77.50) | 15.50  (10.00, 29.75) | 0.001 | 53.00  (29.50, 87.00) | 19.50  (13.00, 40.75) | ＜ 0.001 |
| CRP (mg/L) | 23.10  (8.91, 61.40) | 3.43  (1.73, 11.78) | ＜ 0.001 | 22.10 (5.09, 73.85) | 3.29 (2.83, 6.92) | ＜ 0.001 | 22.50  (7.46, 64.15) | 3.43  (1.95, 8.32) | ＜ 0.001 |
| VEGF (mg/ml) | 624.40(335.20, 1029.50) | 340.70(217.00, 704.90) | 0.027 | 674.05(412.25, 983.58) | 408.40(287.20, 570.90) | 0.027 | 653.80(360.30, 1013.10) | 403.75 (285.55, 636.25) | 0.002 |
| CD19^+^ B cell (cells/μl) | 160.73 (88.89, 250.84) | 178.10(131.97, 296.53) | 0.289 | 142.95 (78.75, 259.08) | 220.00(122.24, 366.08) | 0.049 | 151.98 (84.88, 251.00) | 216.00 (122.94, 313.83) | 0.030 |
| NK cell (cells/μl) | 227.67 (142.54, 328.39) | 282.09(166.83, 372.43) | 0.400 | 199.00 (110.43, 297.06) | 297.00(206.06, 430.58) | 0.022 | 224.40(117.97, 317.54) | 284.82 (183.24, 393.30) | 0.020 |
| CD4^+^ T cell (cells/μl) | 695.81 (425.73, 991.97) | 739.39(610.60, 978.83) | 0.141 | 701.90 (441.05, 982.33) | 781.12(418.75, 1135.44) | 0.581 | 701.00(436.24, 990.76) | 739.39 (566.56, 1088.77) | 0.159 |
| CD8^+^ T cell (cells/μl) | 428.98 (307.91, 584.05) | 444.57(270.01, 657.95) | 0.840 | 432.50 (285.68, 591.41) | 406.95(322.17, 567.75) | 0.849 | 431.98(298.44, 586.06) | 436.57 (303.63, 603.07) | 0.966 |
| Th1 cell (cells/μl) | 124.45 (71.07, 193.74) | 144.60 (93.67, 209.85) | 0.445 | 60.06 (26.82, 127.93) | 78.77 (33.70, 192.86) | 0.505 | 98.88 (46.14, 179.90) | 124.71 (52.61, 208.12) | 0.390 |
| Th2 cell (cells/μl) | 6.77 (4.28, 10.27) | 7.58 (5.32, 12.71) | 0.217 | 7.89 (4.38, 11.73) | 7.18 (4.76, 19.09) | 0.513 | 7.05 (4.35, 11.28) | 7.46 (5.13, 14.15) | 0.190 |
| Th17 cell (cells/μl) | 7.00 (3.96, 12.59) | 11.76 (7.75, 16.00) | 0.021 | 6.30 (3.57, 11.92) | 8.23 (3.25, 13.30) | 0.910 | 6.70 (3.89, 12.20) | 9.91 (4.79, 14.99) | 0.083 |
| Treg cell (cells/μl) | 21.13 (14.62, 30.47) | 25.52 (20.68, 37.20) | 0.080 | 23.95 (16.79, 41.68) | 35.36 (21.56, 53.75) | 0.104 | 22.51 (15.15, 33.06) | 29.06 (21.37, 45.44) | 0.019 |

ACPA, anticitrullinated peptide antibody; RA, rheumatoid arthritis; DAS28, disease activity score using 28 joint counts; MMP-3, matrix metalloproteinase-3; ILD, interstitial lung disease; CVD, cardiovascular disease; WBC: white blood cells; ESR, erythrocyte sedimentation rate; CRP, C-reactive protein; VEGF, vascular endothelial growth factor.

(A)


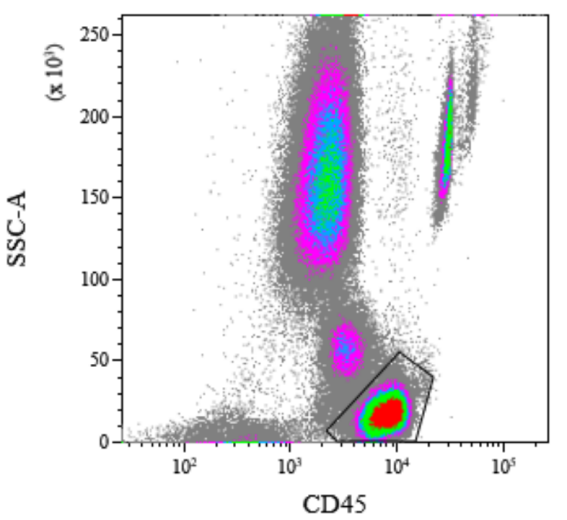


(B)


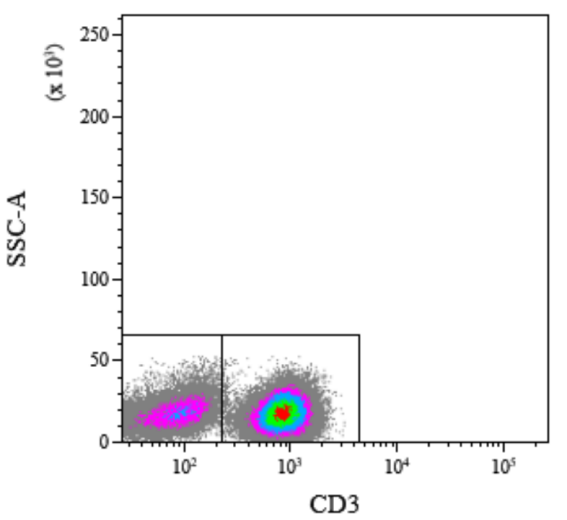


(C) (D)


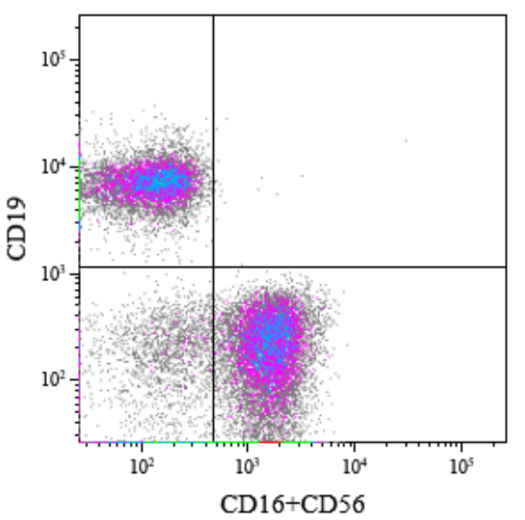

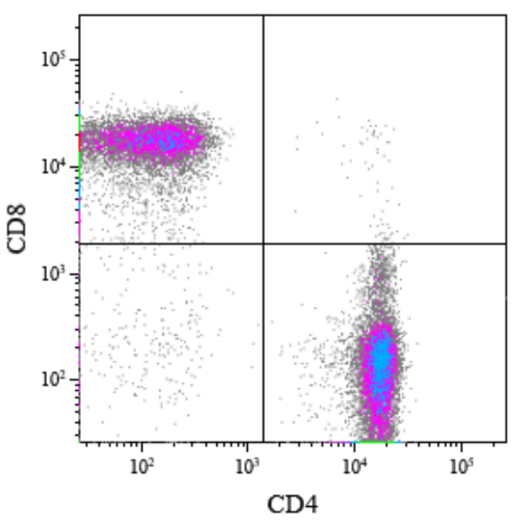


**Supplementary Figure S1a.** Representative flow cytometry analysis of lymphocyte subsets in the peripheral blood of RA patients and healthy controls. (A) (B) The gates include lymphocytes (CD45^+^) and T cells (CD3^+^). (C) The figure showed the regions of B (CD3^-^/CD19^+^) and NK (CD3^-^/CD16^+^/CD56^+^) cells which were from lymphocyte gate. (D) CD4^+^T and CD8^+^T cells that contained in CD3^+^T cells were gated by flow cytometry.

(A)


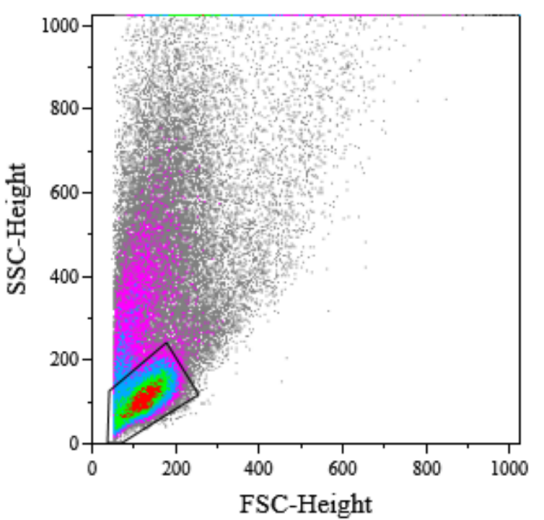


(B)


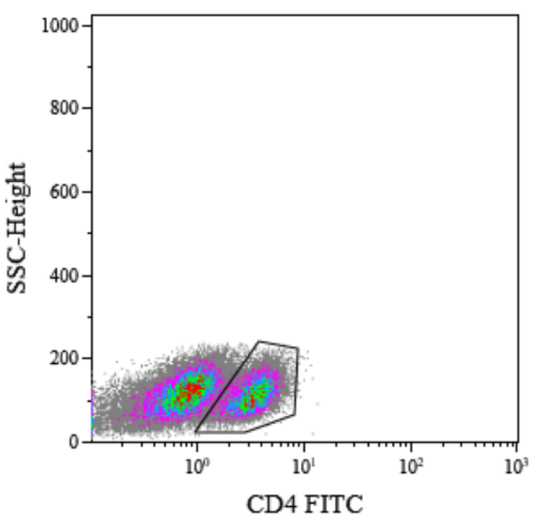


(C) (D) (E)


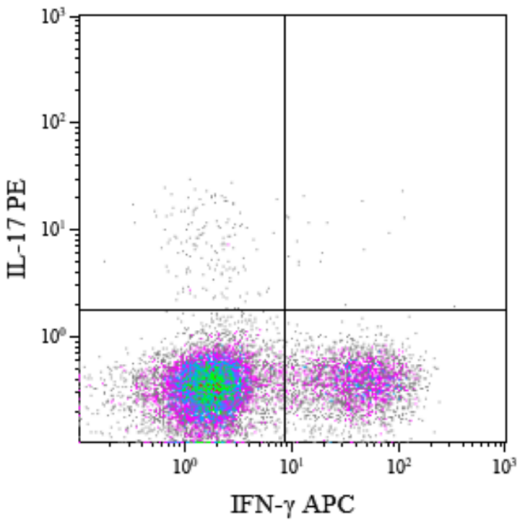

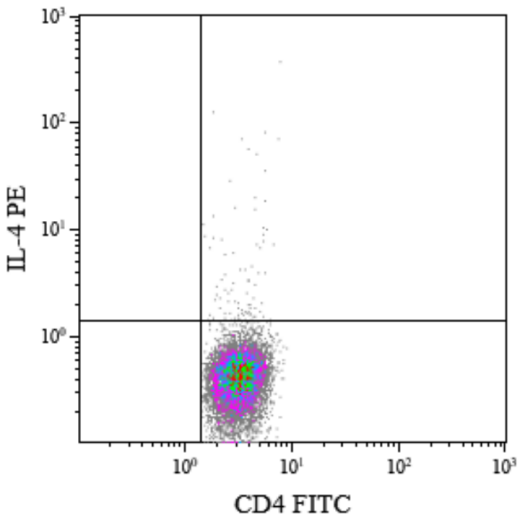

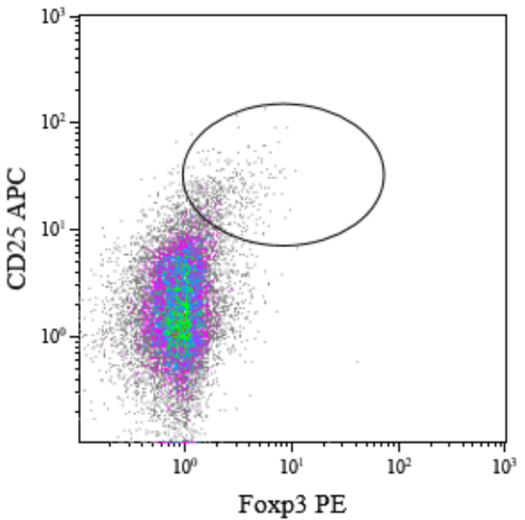


**Supplementary Figure S1b.** Representative flow cytometry analysis of CD4^+^ T cell subsets in the peripheral blood of RA patients and healthy controls. (A) (B) The gates include lymphocytes and CD4^+^ T cells. (C) The figure showed the regions of Th1 (IFN-γ^+^) and Th17 (IL-17^+^) cells which were from CD4^+^T cells gate. (D) (E) Th2 (IL-4^+^) and Treg (CD25^+^Foxp3^+^) cells that contained in CD4^+^T cells were gated by flow cytometry.


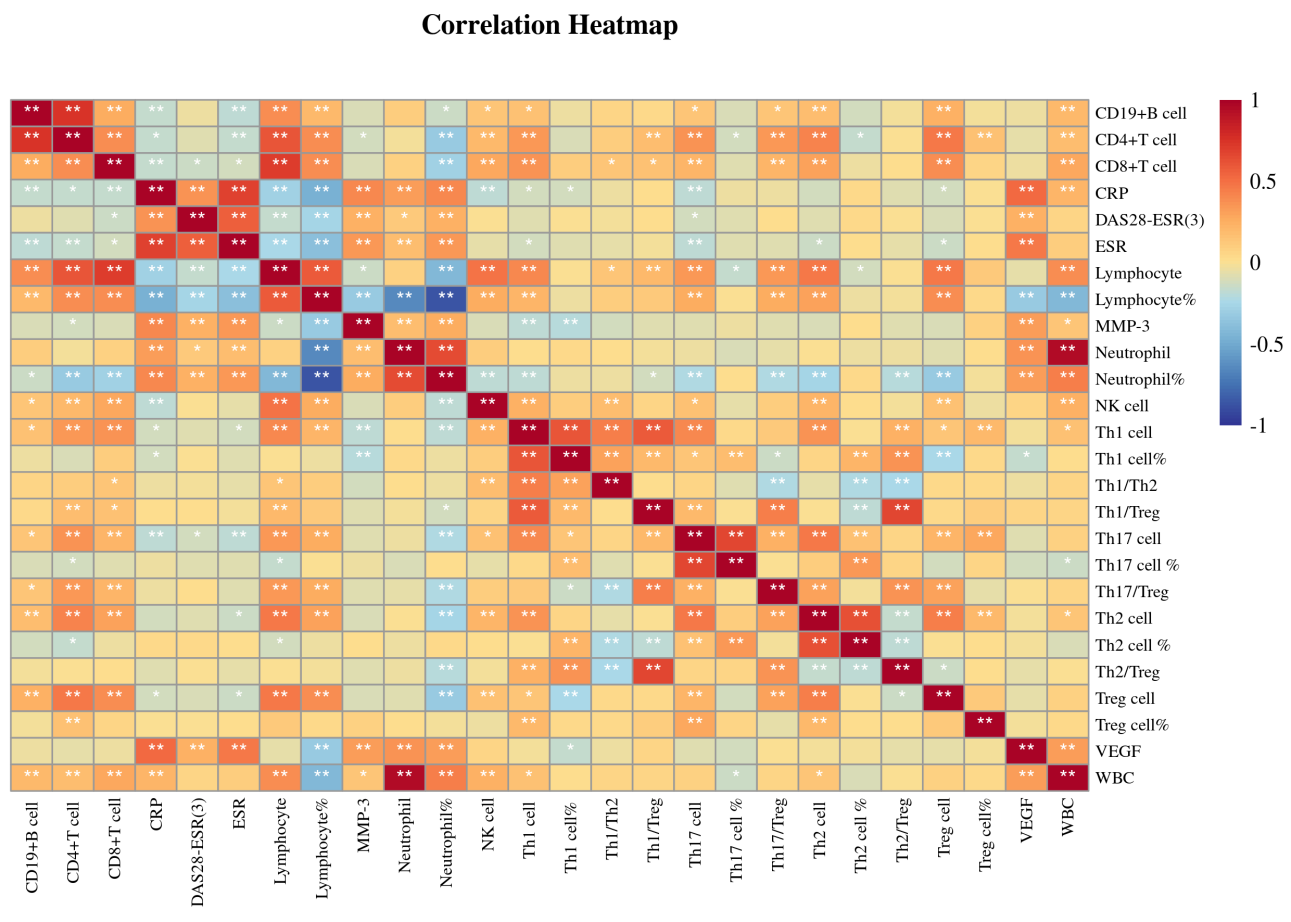


(A)

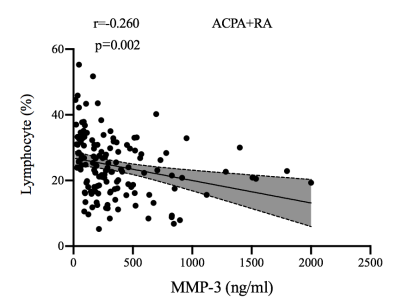


(B)

**
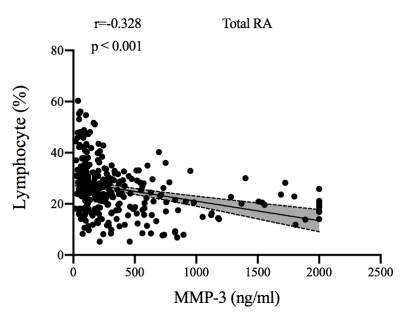
**

(C)

**Supplementary Figure S2.** Correlations between indicators in total RA. (A) Heat map showing the correlation of inflammatory markers, lymphocyte subsets and other indicators in all RA patients. (B) (C) Correlation analysis between MMP-3 and some indexes in ACPA^+^ and total RA patients. **p* < 0.05, ***p* < 0.01.


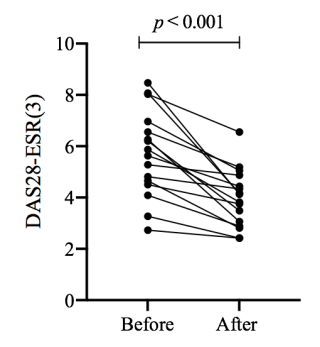

(A)


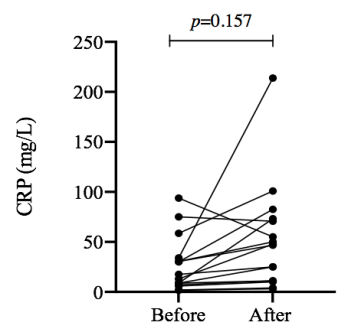

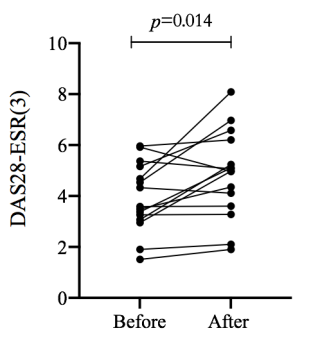


(B)

**Supplementary Figure S3.** The change of MMP-3, ESR, CRP, DAS28-ESR(3), CD4^+^ T cells, CD8^+^ T cells, Th17cells and Treg cells in patients with RA. (A) The changes of various markers in the improvement group. (B) The changes of various markers in progression group.
